# Immunohistochemical Comparison of Dopamine-2 Receptor Expression in Resistant and Non-Resistant Prolactinomas

**DOI:** 10.3390/jcm14207344

**Published:** 2025-10-17

**Authors:** Ilana Ramer Bass, Julia Ferreira de Carvalho, Melissa Umphlett, William Shuman, Alexander Kirschenbaum, Emily Milgrim, Lucas Milgrim, Joshua Bederson, Kalmon Post, Raj Shrivastava, Alice C. Levine

**Affiliations:** 1 Division of Endocrinology, Diabetes and Bone Disease, Department of Medicine, Icahn School of Medicine at Mount Sinai, New York, NY 10029, USA; 2 Department of Pathology, Icahn School of Medicine at Mount Sinai, New York, NY 10029, USA; 3 Department of Neurosurgery, Icahn School of Medicine at Mount Sinai, New York, NY 10029, USA; 4 Department of Urology, Icahn School of Medicine at Mount Sinai, New York, NY 10029, USA

**Keywords:** prolactinoma, dopamine agonist, resistance, D2 receptors

## Abstract

**Background:** Dopamine agonists (DAs) are first-line therapy for prolactin-secreting pituitary adenomas; however, a small proportion of tumors are resistant. Previous reports suggested that reduced D2R mRNA expression might cause resistance. This study aimed to determine if resistant prolactinomas express D2R protein. We also explored a role of estrogen receptor alpha (ERα) expression in DA resistance. **Methods:** We retrospectively selected 15 tumor specimens from 13 total patients (8 controls from 8 patients, 7 from 5 resistant patients) with resected lactotroph cell-type tumors. We reviewed age at diagnosis, tumor size, initial prolactin level, medical treatment, and reason for surgery. Immunohistochemistry was performed for D2R, prolactin, and ERα protein expression. **Results:** D2R expression was positive in seven of eight controls vs. two of seven in resistant tumors (*p* = 0.02). ERα expression did not significantly correlate with DA resistance. The two D2R expressing resistant tumors were ERα negative and both derived from a pre-pubertal female, supporting prior reports suggesting ERα may modulate DA therapy response. **Conclusions:** Our study introduces a reproducible method for assessing D2R protein expression in prolactinomas using commercially available D2R antibodies. Our findings align with current evidence indicating that lack of D2R expression, previously indicated by decreased mRNA levels, is common in DA-resistant prolactinomas and provide a basis for discontinuation of DA therapy to avoid potential harm to these patients.

## 1. Introduction

Dopamine agonists (DAs) are the first-line therapy for prolactin-secreting pituitary adenomas as they are effective in improving symptoms and reducing tumor burden [1]. Bromocriptine and cabergoline work by binding and activating the dopamine-2 receptor (D2R) on the pituitary lactotroph cell membrane [2].

The reported prevalence of DA resistance is approximately 20–30% with bromocriptine and 10% with cabergoline [3,4]. A recent consensus statement from the Pituitary Society defines DA resistance as a lack of normalization of prolactin serum levels or lack of relevant mass shrinkage (≥30% reduction in maximum diameter) when treated with standard dopamine agonist doses (7.5–10 mg/day of bromocriptine or 2.0 mg/week of cabergoline) for at least 6 months [4,5,6].

It is important to accurately identify patients who will respond to DA therapy, as DAs have potential adverse effects, including postural hypotension, mental fogginess, and, less commonly, symptoms of psychosis and impulse control disorders [7,8]. At very high doses used to treat patients with Parkinson’s disease, DAs increase the risk of cardiac valve regurgitation [7,9].

In growth hormone-secreting adenomas it has been reported that determination of somatostatin receptors (SSTRs) by immunohistochemistry (IHC) can predict treatment response to somatostatin receptor ligands [10]. Previous reports have proposed a reduction in D2R expression as a molecular mechanism for DA resistance, but these studies primarily measured D2R mRNA [11,12]. The primary objective of this study was to determine if resistant prolactinomas express D2R protein as assessed by immunohistochemistry, with the aim of avoiding unnecessary use of high-dose dopamine agonists in D2R negative prolactinomas. Additionally, we investigated whether estrogen receptor alpha (ERα) expression modulated the response to dopamine agonists, as demonstrated in previous reports [13].

## 2. Materials and Methods

### 2.1. Study Design

#### 2.1.1. Study Design—Patient and Tumor Characteristics

This retrospective study was conducted at an academic quaternary-care center and approved by the Institutional Review Board of Mount Sinai Hospital. The study included thirteen patients with surgically resected lactotroph cell-type tumors.

Patients were classified as resistant if they failed to achieve normal prolactin levels and failed to achieve tumor size reduction of at least 50% with maximal conventional medication dosages (as defined above).

Simultaneously, a chart review of the Electronic Medical Record was conducted to gather information about age at diagnosis, tumor size, initial prolactin level, medical treatment, reason for surgical resection, and whether recurrence occurred.

#### 2.1.2. Study Design—IHC

The tumors were classified according to the World Health Organization classification of pituitary tumors (3rd edition) [14]. Multiple unstained 5 μm thick sections were cut for IHC analysis from 15 paraffin blocks, and the following markers were assessed: D2R, prolactin, and ERα.

All steps for IHC were carried out in a humidified chamber. Tissues were formalin-fixed and paraffin-embedded. Sections were deparaffinized, rinsed in graded alcohol, rinsed in water, and treated with 3% hydrogen peroxide in 100% methanol for 10 min. Sections were then rinsed in water and placed in an antigen retrieval solution (1% citrate buffer), which was carried out in a pressure cooker. Following antigen retrieval, the sections were blocked for 30 min with normal goat serum and then incubated overnight at 4°C with D2R, prolactin, and ERα antibodies (see Table 1).

After incubation, the sections underwent 3 PBS washes, then incubated in biotinylated goat anti-rabbit (for D2R) or anti-mouse (for prolactin, ERα) for 30 min, followed by 3 PBS washes. Next, sections were incubated with streptavidin-horseradish peroxidase for 30 min. Afterwards, the sections were washed in 0.5% Triton X 100 for 30 s followed by the application of the chromagen, diaminobenzidine, for 1–2 min in the dark. The sections were counterstained with hematoxylin. Positive and negative controls were run in parallel.

A board-certified neuropathologist (MU) reviewed the slides in a blinded fashion. Immunostaining was scored as positive or negative. Tumors were evaluated for receptor expression based on the percentage of stained cells and the intensity of staining. Percentage of stained cells was scored as follows: 0 (<25% stained cells), 1 (25–50%), 2 (50–75%), or 3 (75–100%). Intensity of staining was scored as follows: 0 (negative), 1 (weak), or 2 (intermediate to strong). To obtain a total score, the percentage of stained cells and intensity score were added together. Both intracytoplasmic and membrane-bound D2R immunopositivity were considered as positive.

### 2.2. Statistical Analysis

Quantitative variables were expressed as median (minimum–maximum range) due to large data ranges. Absolute frequencies were reported for categorical variables. Comparisons between nominal groups and continuous values were performed using chi-squared tests and Mann–Whitney tests, respectively. A *p*-value of 0.05 was considered significant. Unadjusted odds ratios were calculated using logistic regression in STATA 19.

## 3. Results

In our study, 13 patients were included. However, due to two patients undergoing two surgeries for resistant prolactinomas, the sample size comprised 15 specimens: 7 from five resistant patients and 8 sections from the eight control patients. Controls were prolactinomas resected for reasons other than treatment resistance including intolerance due to side effects or patient preference.

The resistant group had two men and three women, while the control group consisted of four women and four men. The median age at diagnosis was similar in both groups (35 vs. 29 years for resistant and control groups, respectively). The median baseline prolactin level was higher in the resistant group (1014 ng/mL, range 160–11,231 ng/mL) compared to the control group (326 ng/mL, range 140–4180 ng/mL) (*p* = 0.16, U = 13). For patients with two samples, PRL levels at time of diagnosis of both tumors were used. Tumor size was larger in the resistant group, with a median size of 2.13 cm (range 1.4–2.9 cm) compared to 1.50 cm (range 0.8–3.3 cm) in the control group (*p* = 0.96, U = 55). The median maximal weekly cabergoline dose given in the resistant group was 3 mg (range 2–7 mg) compared to 2 mg (range 0.25–7.5 mg) in the controls (see Supplementary Materials Table S1).

Age at diagnosis was not significantly associated with D2R expression (OR = 0.989, 95% CI 0.927–1.06, *p* = 0.756). Similarly, sex did not show a significant association (OR = 0.667, 95% CI 0.069–6.41, *p* = 0.725). Initial prolactin levels were also not associated with D2R expression (OR = 1.00, 95% CI 0.999–1.001, *p* = 0.476). Finally, largest tumor dimension did not significantly predict the presence of D2R (OR = 0.655, 95% CI 0.106–4.04, *p* = 0.649).

### Immunohistochemical Expression

**Figure 1:**
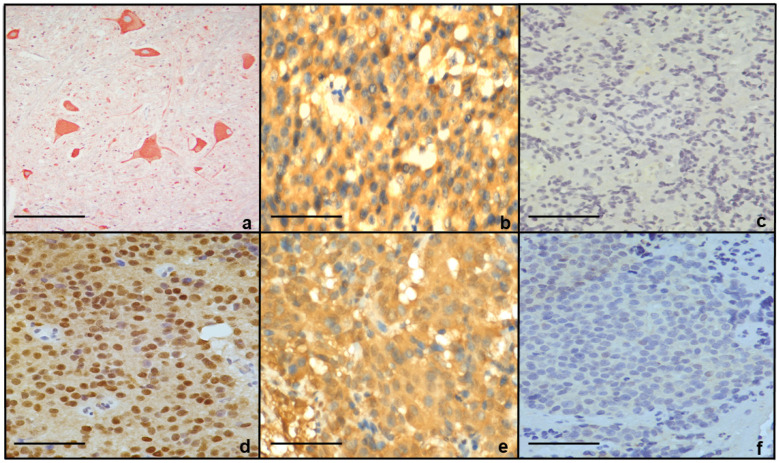
D2R and Erα receptor IHC of (**a**) D2R staining neurons positive in normal spinal cord (200X); (**b**) 28-year-old male (non-resistant) with tumor cells positive for D2R (400X); (**c**) 34-year-old male (resistant) with tumor cells negative for D2R (400X); (**d**) 31-year-old female (non-resistant) with tumor cells positive for ER (400X); and (**e**,**f**) 11-year-old pre-pubertal girl with a resistant tumor whose tumor cells were positive for D2R and negative for Erα ((**e**) 400X, (**f**) 400X)). Scale bar, 50 μm.

PRL was expressed by all tumors. D2R expression was positive in seven out of eight controls (88%) vs. two out of seven (29%) in resistant tumors (*p* = 0.02, chi square = 5.40). Erα positivity did not correlate significantly with DA resistance (positive in 43% of resistant vs. 63% of controls, *p* = 0.44, Chi square = 0.579) (Figure 1).

The two resistant tumors that retained D2R expression were both derived from a single patient, an 11-year-old pre-pubertal girl who underwent two resections for a resistant prolactinoma. Although her two tumors were positive for D2R they were negative for Erα protein expression.

## 4. Discussion

Several recent studies have focused on investigating resistant prolactinomas to predict which patients are likely to respond to treatment, thereby sparing those who would not from potential adverse effects of dopamine agonist therapy. Our primary aim was to evaluate the protein expression of D2R by IHC in resistant prolactinomas. We observed a statistically significant difference in D2R expression between resistant (71% negative) and control (12% negative) prolactinomas. This finding aligns with studies demonstrating a correlation between D2R gene expression and responsiveness of prolactinomas to DA [15,16,17]. Similarly, Pellegrini et al. demonstrated a reduced density of dopaminergic binding sites labeled by [3H] spiroperidol in resistant prolactinomas [18]. Notably, Tang et al. published a pilot study that used F-fallypride PET/MR to determine D2R expression, enabling them to predict dopamine agonist resistance [19]. Peng et al. demonstrate the importance of abnormal cholesterol metabolism in reducing membrane localization of D2R in resistant prolactinomas and how interfering with the cholesterol metabolism may help attenuate the reduced membrane localization of D2R and attenuate DA resistance [20]. Trouillas et al. underscored the need for D2R profiling by IHC but in 2019 no specific commercial antibodies were available [21]. Recently, Rajan et al. used IHC to predict the response to cabergoline therapy in patients with somatotroph adenomas [22]. Our study provides a feasible approach using IHC to assess for D2R expression in lactotroph adenomas.

Estrogens have been shown to modulate the inhibitory effect of dopamine on prolactin gene transcription by altering the expression of different D2R isoforms on lactotrophs [23]. Given this information, our secondary aim was to compare ERα expression in both groups to investigate its potential role in dopamine agonist resistance. We found that ERα expression did not correlate with DA resistance in our study. This finding is consistent with Wu et al. and Cristina et al., who reported no significant difference in ERα mRNA expression between responsive and resistant prolactinomas [24,25].

However, it is worth noting that among the resistant tumor samples, the two specimens that retained D2R expression were both derived from an 11-year-old pre-pubertal girl who underwent two distinct surgeries. We hypothesize that DA resistance in this case may be explained by the lack of ERα expression in the tumor, rendering the tumor insensitive to DA even in the presence of D2R protein. Radl et al. previously showed that DA can induce apoptosis in lactotrophs *only* in the presence of E2, hypothesizing that estrogens may exert a permissive role on the apoptosis induced by DA [13].

There are several limitations to our study that should be acknowledged. Firstly, our sample size was relatively small, and the number of cases included might have influenced the statistical power of our analysis and hindered our ability to detect significant associations. Secondly, the absence of standardized diagnostic protocols for defining the duration of therapy in order to classify resistant prolactinomas introduces variability in the patient population and treatment outcomes. Furthermore, the retrospective design of our study also limited the data available. Lastly, the control group used in our study was heterogeneous in terms of the reason for resection. This might have introduced confounding factors and could have impacted the expression of the receptors being investigated. Additionally, it cannot be excluded that protein expression of the receptors may have been affected by exposure to dopamine agonist therapy.

In summary, our study introduces a straightforward and reproducible method for assessing D2R protein expression in prolactinomas through the utilization of commercially available D2R antibodies. Our findings align with current evidence indicating that lack of D2R expression, previously indicated by decreased mRNA levels, is common in DA-resistant prolactinomas and provides a basis for the discontinuation of DA therapy in D2R negative prolactinomas to avoid possible side effects. Additionally, it is plausible that ERα modulates the response to DA therapy and that the absence of ERα in one young patient rendered her tumor resistant even in the presence of D2R protein expression.

## Figures and Tables

**Table 1:** Illustrating IHC proteins, antibodies used, dilution needed, and species of antibodies.

Peptide/Protein	Antibody Manufacturer	Catalog Number	Species Raised in (Mono- or Polyclonal)	Dilution	RRID Number
D2R	Novus Bio	NLS1403	Rabbit; polyclonal	1:1000	AB_523304
Prolactin	BioGenex	AM978-5M	Mouse; monoclonal	Ready to use	AB_3065212
ERα	Dako	M7047	Mouse; monoclonal	1:100	AB_2101946

## Data Availability

Most raw data supporting the conclusions of this article is available in the article, any additional data will be made available by the authors, without undue reservation.

## Supplementary Materials

The following supporting information can be downloaded at: https://www.mdpi.com/article/10.3390/jcm14207344/s1, Table S1: Patient and Tumor Characteristics.
