# Supplementary material for: Immunohistochemical Comparison of Dopamine-2 Receptor Expression in Resistant and Non-Resistant Prolactinomas
Source: J Clin Med. 2025 Oct 17;14(20):7344. doi: 10.3390/jcm14207344 (PMC12565039; doi:10.3390/jcm14207344)
Supplement: Supplementary file 1 [file jcm-14-07344-s001.zip › jcm-3872560-supplementary.pdf]

Supplementary Table S1. Patient and Tumor Characteristics.

**Patient characteristics**	**Resistant (R)**	**Control (C)**
Patients (n=13)(%)	5 (38)	8 (62)
Women	3	4
Men	2	4
Specimens (n=15)	7	8
Median age at diagnosis (range 11-80) (years) (p=0.38, U value 13.5)	34.5	29
Median baseline prolactin[a] (range 11231-140) (ng/mL) (p=0.16; U value 13)	1014	326
Median tumor size (range 0.8-3.3)[b] (largest diameter (cm) (p=0.96, U value 55)	2.13	1.50
Median maximal cabergoline dose given (range)[c](mg) (p=0.47, U value 8.5)	3 (2-7)	2 (0.25-7.5)

[a] Data unavailable for 1 patient, [b] Data unavailable for 2 specimens, [c] Data unavailable for 3 tumors
